# Perceived emotional states mediate willingness to buy from advertising speech

**DOI:** 10.3389/fpsyg.2022.1014921

**Published:** 2023-01-09

**Authors:** Mizuki Nagano, Yusuke Ijima, Sadao Hiroya

**Affiliations:** ^1^NTT Human Informatics Laboratories, NTT Corporation, Yokosuka, Japan; ^2^NTT Communication Science Laboratories, NTT Corporation, Atsugi, Japan

**Keywords:** willingness to buy, advertising speech, emotional states, SOR theory, PAD model, age difference, gender difference

## Abstract

Previous studies have shown that stimulus-organism-response (SOR) theory can well explain the willingness to buy from stores, products, and advertising-related stimuli. However, few studies have investigated advertising speech stimulus that is not influenced by visual design. We examined whether SOR theory using emotional states can explain the willingness to buy from advertising speech stimulus. Participants listened to speech with modified speech features (mean F0, speech rate, and standard deviation of F0) and rated their willingness to buy the advertised products and their perceived emotional states (pleasure, arousal, dominance). We found that emotional states partially mediate the influence of speech features on the willingness to buy. We further analyzed the moderating effects of listeners' attributes and found that listeners' gender and age group moderated the relationship between speech features, emotional states, and willingness to buy. These results indicate that perceived emotional states mediate the willingness to buy from advertising speech.

## 1. Introduction

Retailers and manufacturers always make an effort to attract as many customers as possible and increase sales. They pay attention to many factors regarding their stores and products, e.g., store environment, layout, product packaging, and advertising. These factors make customers interested in the product and increase their willingness to buy.

It is well known that atmosphere, crowding, background music, image, glossiness, haptic features, and speech influence the willingness to buy (Kotler, 1973; Turley and Milliman, 2000; Krishna, 2012). In consumer behavior research, stimulus-organism-response (SOR) theory as a hierarchical model was proposed to explain the willingness to buy from these stimuli and is better than directly explaining the willingness to buy from the stimuli (Mehrabian and Russell, 1974). It is suggested that high-level processing in the brain may be involved in determining the willingness to buy from stimuli. In SOR theory, a model that uses pleasure, arousal, and dominance (PAD) emotional states as an organism is called the PAD model (Donovan and Rossiter, 1982). This model has successfully been used to explain the effects of store-related stimuli, such as crowding and background music, and product-related stimuli, such as glossiness and haptic features, on the willingness to buy (Mari and Poggesi, 2013; Briand Decré and Cloonan, 2019). There has also been research on the effect of advertising-related stimuli, such as copy, image, video, and speech, on the willingness to buy. For example, previous studies reported on the copy and image of catalogs and video advertisements (Fiore, 2002; Wu and Chen, 2016). There is a consistently positive correlation between the pleasure emotional state and willingness to buy (Donovan et al., 1994; Anwar et al., 2020).

Speech is also used as advertising-related stimuli, especially for effective broadcasting advertising. However, there is not a very consistent relationship between speech features and the willingness to buy. For example, Chattopadhyay et al. have showed that a faster speech rate and low pitches have influenced the willingness to buy (Chattopadhyay et al., 2003), while Peterson et al. have showed that there was no correlation between mean F0 and SD F0 of salespeople and performance (Peterson et al., 1995). Since speech is closely related to emotional states and known to affect the perceived impression or behavior of the listener (Schröder, 2001; Tsantani et al., 2016; Li and Akagi, 2018), such a nonlinear relationship between speech features and the willingness to buy may be explained using a hierarchical model. In fact, in their study on the willingness to buy, Poon et al. did not use the PAD model but investigated the influence of the differences in pause length of male and female speech on the willingness to buy through a perceived personality state (Poon et al., 2018). However, there have been few studies on the PAD model and taking into account speech as a stimulus, including the willingness to buy as a response.

We examined whether the PAD model can explain the willingness to buy from the speech stimulus of electric appliance advertisements spoken by male and female professional narrators. To verify the effect of the difference in the speech features, the speech was generated with the converted static and dynamic features of F0 and speech rate, which have been used to study speech features and emotional states (Schröder, 2001). These speech features can be manipulated with simple speech signal processing. Large-scale subjective evaluations were conducted *via* crowdsourcing. Participants were asked to evaluate their emotional states, i.e., PAD and their willingness to buy when listening to an advertising speech. We conducted an analysis of variance (ANOVA), path analysis, mediation analyzes, and moderated mediation analysis using the obtained data of the speech features of speech stimuli, emotional states, and the willingness to buy.

## 2. Stimulus-organism-response (SOR) theory and hypotheses

**Figure 1:**
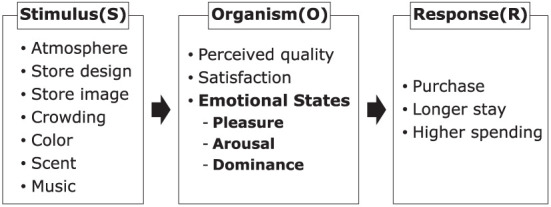
Stimulus-organism-response (SOR) theory.

SOR theory (Mehrabian and Russell, 1974) comprises three dimensions: stimulus (S), organism (O), and response (R) (Figure 1). Stimulus incorporates all external environmental factors in the store such as atmosphere (Donovan et al., 1994), design (Jang et al., 2018; Nusairat et al., 2020), brand image (Simanjuntak et al., 2020), crowding (Anninou et al., 2018), color, scent, and music (Roschk et al., 2017). These stimuli stimulate various consumer responses. In previous studies, the responses were investigated using perceived quality (Nusairat et al., 2020), satisfaction (Roschk et al., 2017), and emotional states (Anwar et al., 2020). These consumer responses result in approach or avoidance behavior. Approach behavior refers to a positive attitude toward the environment, such as staying in a place. In contrast, avoidance behavior refers to a negative attitude toward the environment, such as escaping from a place. In consumer behavior research, approach or avoidance behavior is confirmed with indicators such as purchase, longer stay time (Jang et al., 2018) and higher spending (Milliman, 1982).

The PAD model specifically focuses on emotion as the organism in SOR theory. There are three emotional states: PAD (Donovan and Rossiter, 1982). Pleasure refers to the degree of feeling joy, satisfaction, and happiness with a situation. Arousal refers to the degree of feeling excited, passionate, and active about the situation. Dominance refers to the degree of feeling that an individual has influence over a situation and can control it. The PAD model has been verified with various external stimuli, and the evidence from previous studies has shown that this model reflects the relations among these stimuli, consumers' emotional states, and consumer' responses (Donovan and Rossiter, 1982; Milliman, 1982; Donovan et al., 1994; Mari and Poggesi, 2013; Briand Decré and Cloonan, 2019; Anwar et al., 2020). We expect the PAD model to also explain the willingness to buy caused by speech stimuli.

**Figure 2:**
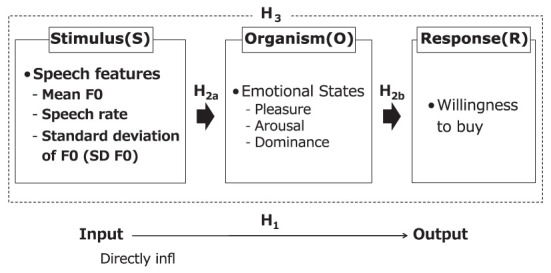
Conceptual model (*H*_1_–*H*_3_).

Thus, the paper attempts to verify the conceptual model (Figure 2) and the following four hypotheses:

*H*_1_: Speech features directly influence the willingness to buy (S-R). No specific hypothesis about speech features is given because of the lack of consistent results in previous studies.

*H*_2*a*_: Speech features influence emotions (S-O).

*H*_2*b*_: Emotional states influence the willingness to buy (O-R). In particular, when pleasure increases, the willingness to buy increases.

*H*_3_: Emotional states mediate the influence of speech features on the willingness to buy (SOR).

The main purpose of this study was to verify *H*_3_, and the verification of *H*_1_ and *H*_2_ is to confirm the preconditions of *H*_3_.

However, the effects of emotional mediating on the willingness to buy from advertising speech may vary depending on attributes such as age and gender of the listener. For example, Schirmer et al. (2005) reported that female participants were more capable of recognizing emotions than male participants. Coley and Burgess (2003) report that the effect of emotional states on impulse buying tends to be more evident for females than with males. Regarding age differences, it is well known that older people have a tendency to preferentially process positive stimuli relative to negative stimuli, called the positive effect (Reed and Carstensen, 2012), and that hearing loss with age (Dupuis and Pichora-Fuller, 2015). Older people have also been reported to have lower emotion recognition abilities than younger adults (Lima et al., 2014; Schmidt et al., 2016). Therefore, the relationship between advertising speech, emotional states, and the willingness to buy may be moderated by the attributes of the listener. At least, it is expected that the attributes of the listener will influence the relationship between emotion and the willingness to buy, which is consistent with previous studies.

**Figure 3:**
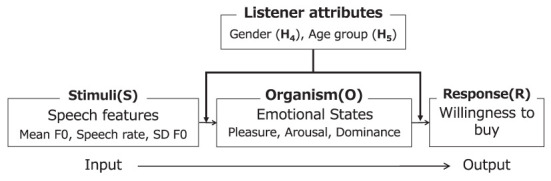
Conceptual model (*H*_4_, *H*_5_).

We further attempted to verify the conceptual model (Figure 3) and the following two hypotheses:

*H*_4_: The relationship between advertising speech and emotional states or between emotion and the willingness to buy is moderated by the gender of the listener.

*H*_5_: The relationship between advertising speech and emotional states or between emotion and the willingness to buy is moderated by the age group of the listener.

## 3. Subjective evaluation

### 3.1. Speech material

Four sentences of read speech stimuli spoken by one Japanese male and one Japanese female professional narrators were used. All sentences were gathered from the Web and intended to promote electrical appliances that customers cannot recognize much of a difference between brands for products (Assael, 1984). Since all of the advertising sentences we used were about older products, we also concluded that listeners are less likely to be influenced in their willingness to buy by the advantage of the products advertised in the sentences. Advertising speech for four electrical appliances was used: air conditioner, washing machine, refrigerator, and PC. Sentences translated into English are included in the Supplementary material.

Both narrators spoke the same four sentences. The average F0, standard deviation of F0 (SD F0), sentence duration, and speech rate were 112.87 [Hz], 1.34 [Hz], 15.41 [s], and 6.57 [mora/s] for the male speech and were 255.53 [Hz], 1.30 [Hz], 14.87 [s], and 7.02 [mora/s] for the female speech, respectively. The sampling frequency of the recorded speech was 22.05 [kHz]. Each phoneme boundary was manually segmented. We attempted to minimize the difference in SD F0, pause duration, and speech rate between male and female speech by recording speech, but in addition to the mean F0, there was a significant difference in the speech rate between genders [mean F0: *t*_(6)_ = 25.61, *p* < 0.05, SD F0: *t*_(6)_ = 0.89, *p* = 0.41, speech rate: *t*_(6)_ = 5.16, *p* < 0.05].

To verify the influence of speech features on emotional states and the willingness to buy, we manipulated the mean F0 [Hz], SD F0, and speech rate [mora/s] of the original speech. Mean F0 was converted by a factor of 0.94 (low) or 1.06 (high) to the average F0 of the sentence using WORLD (Morise et al., 2016), and speech rate was converted by a factor of 1.12 (slow) or 0.89 (fast) by PICOLA (pointer interval controlled overlap and add) (Morita and Itakura, 1986). The conversion rate for mean F0 was determined on the logarithmic axis to take into account human auditory characteristics. SD F0 was converted by a factor of 1.50 (more variation) or 0.67 (less variation) (Fukuoka et al., 2016). These parameters were determined through preliminary experiments so that the converted speech would not sound unnatural compared with human speech. To reduce the influence of sound quality deterioration due to speech modification mentioned above, all stimuli used in the subjective evaluation were analyzed and synthesized using WORLD: a process of analysis-by-synthesis by WORLD was also conduced even for the original speech used as a baseline. We used 27 types of speech stimuli per sentence in the combination of mean F0, speech rate, and SD F0 [3 (mean F0: low vs. original vs. high) × 3 (speech rate: slow vs. original vs. fast) × 3 (SD F0: large vs. original vs. small)]. Pause duration was matched by adjusting the silent sections between male and female speech. The average intensity level of all stimuli was 62 dB.

### 3.2. Participants

The participants were 457 native Japanese speakers. They were recruited *via* a crowdsourcing service and participated in the experiment on their judgment. Fifty-seven participants had to be excluded from the analysis due to incomplete answers. The remaining 400 participants were available for analysis (247 males and 153 females, mean age = 42.48 years, SD = 14.29, range = 21–70). The participants' age group and gender are shown in Table 1. All participants were paid after their completion of the experiment.

**Table 1:** Participants' age groups and gender.

	**20s**	**30s**	**40s**	**50s**	**60s**	**Total**
Male	48	49	45	52	53	247
Female	35	43	33	20	22	153
Total	83	92	78	72	75	400

### 3.3. Procedure

Each participant listened to the speech of either a male or female narrator. Two of the four sentences (54 stimuli) were selected for each participant, taking counterbalance into account. The experiment was conducted in a browser *via* crowdsourcing. The participants listened to the speech stimuli only once, rated the degree of their willingness to buy the advertised product, then rated their perceived emotional states in the browser. The order of evaluated items was designed on the basis of a previous study (Milliman, 1982). The willingness-to-buy response was removed from the display when the participants answered regarding their emotional states. Because of the crowdsourcing experiment, it was difficult to control the playback equipment and presented sound-pressure level among the participants, so they participated at a comfortable volume for them. Note that there is little difference between the results of auditory laboratory experiments and crowdsourcing experiments (Cooke and García Lecumberri, 2021).

We aimed to investigate the effects of changing the speech features of the same speaker's speech on emotional states and willingness to buy. Therefore, the speakers of the advertising speech were limited, but we considered are all combinations of changes in speech features.

The willingness to buy was rated on a 7-point Likert scale (1: not at all willing to buy–7: very willing to buy). Participants were given the following instructions: “You don't really have to think about whether or not to buy it because we want you to evaluate “motivation.” Please answer not how you feel about the manufacturer or brand, but how you feel about the narrator's way of speaking.” The participant's emotional states were rated on a 7-point Likert scale: [pleasure (pleasant–unpleasant), arousal (calm–excited), and dominance (dominant–submissive)]. Participants were given the instructions used in a previous study (Mori et al., 2005) to make it easier to understand these emotional state dimensions (e.g., “Pleasure refers to how good or bad you feel”). Participants answered their age group and gender at the end of the experiment. The experiment lasted about 30 min.

### 3.4. Analysis

For all analyzes, the evaluation values for emotional state and the willingness to buy collected in the experiment were used. The mean F0 [Hz], SD F0 [Hz], and speech rate [mora/s] in the speech interval of each stimulus were used as speech features. Since there were significant differences between the male and female speech in mean F0 and speech rate, which were obtained by subtracting the average values of each were used in the analysis below.

To analyze the direct influence of speech features on the willingness to buy (*H*_1_), a three-way ANOVA was conducted. The significance of the main effects of speech features (mean F0, speech speed, and SD F0) and their interactions were verified. We should confirm that speech features need to influence the willingness to buy before conducting the following path and mediation analyzes.

Path analysis was conducted to examine direct dependencies between speech features and emotions (*H*_2*a*_) and between emotions and willingness to buy (*H*_2*b*_). Path analysis is a commonly used analysis method in consumer-behavior research (Nusairat et al., 2020). It can verify hypothesized models by analyzing direct and indirect relationships between multiple variables. The goodness-of-fit index indicates how well the hypothesized model fits the experimental data. The degree of the effect between variables can be compared using the standardized path coefficient.

Path analysis is insufficient for verifying the importance of considering emotional states to estimate the willingness to buy from speech features. Thus, mediation analysis was used to verify whether emotional states mediate the influence of speech features on the willingness to buy (*H*_3_). With this analysis, it is assumed that a mediator *M* has a potential influence between an independent variable *X* and dependent variable *Y* when *X* affects *Y* (Rijnhart et al., 2021). There should be a causal relationship between *X* and *Y* as well as between *X* and *M* and, *M* and *Y*. This analysis is used to verify how *M* contributes. The effect of *X* on *Y* is called the total effect *t*. The effect of *X* when adjusted for *M* is called the direct effect *d*. The mediating effect *e* is defined as *t*−*d*. Each statistical significance was concluded with 1,000 bootstrap samples. The effect is considered significant if it does not include zero within the 95% confidence interval calculated from the bootstrap samples. When *t* is significant and *d* is not significant, *M* is regarded to be completely mediating the effect of *X* on *Y* and is called “complete mediation.” When both *t* and *d* are significant, *M* is regarded to be partly mediating the effect of *X* on *Y* and is called “partial mediation.”

**Figure 4:**
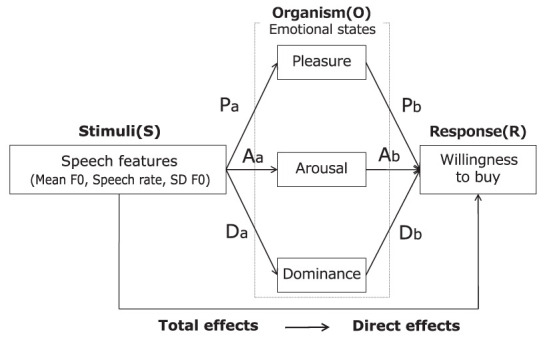
Concept of mediation analysis.

The speech features were entered as independent variables, emotional states as the mediating variable, and the willingness to buy as the dependent variable. In Figure 4, these effects are shown as the following equations.


$$e=t-d=P_{a}\times P_{b}+A_{a}\times A_{b}+D_{a}\times D_{b}$$

Because variables must have causal relationships, only significant paths were used in the analysis on the basis of the results of the path analysis.

We conducted a moderated mediation analysis to examine whether the gender and age group of the listener moderated the pathways shown in Figure 3 in the mediation process of speech features on willingness to buy through emotional states (*H*_4_, *H*_5_; Edwards and Lambert, 2007; Hayes, 2015). We calculated the moderated *e* of speech features on the willingness to buy and the conditional indirect effect at specific levels of the gender or age group when emotional states as a mediator. To avoid the problem of multicollinearity, each variable was centered. In the conditional indirect effect, the representative values of the moderators (e.g., mean, mean±SD, or dummy variable) were selected (Aiken et al., 1991; Kim and Bae, 2019). Each statistical significance was concluded with 1,000 bootstrap samples as with mediation analysis. The effect is considered significant if it does not include zero within the 95% confidence interval calculated from the bootstrap samples.

## 4. Results of applicability of PAD model using advertising speech

### 4.1. Three-way ANOVA

A three-way ANOVA was conducted to analyze the direct influence of speech features on the willingness to buy. Table 2 shows the mean and variance for the evaluation values used in the analysis. There were main effects of mean F0 [*F*_(2, 21, 573)_ = 39.66, *p* < 0.01], speech rate [*F*_(2, 21, 573)_ = 1870.59, *p* < 0.01], and SD F0 [*F*_(2, 21, 573)_ = 529.60, *p* < 0.01]. The first-order interaction effects were observed between speech rate and SD F0 [*F*_(4, 21, 573)_ = 4.55, *p* < 0.01], but not between mean F0 and speech rate [*F*_(4, 21, 573)_ = 2.17, *p* = 0.07] or between mean F0 and SD F0 [*F*_(4, 21, 573)_ = 1.99, *p* = 0.09]. The significant first-order interaction effect was analyzed for simple main effects. The results indicate that each factor was significant. The faster speech rate enhanced the willingness to buy under all SD F0 conditions [under less condition: *F*_(2, 7, 197)_ = 676.30, *p* < 0.01; under original condition: *F*_(2, 7, 197)_ = 686.10, *p* < 0.01; under more condition: *F*_(2, 7, 197)_ = 515.00, *p* < 0.01]. More SD F0 enhanced the willingness to buy under all speech rate conditions [under slow condition: *F*_(2, 7, 197)_ = 130.60, *p* < 0.01; under original condition: *F*_(2, 7, 197)_ = 225.00, *p* < 0.01; under fast condition: *F*_(2, 7, 197)_ = 129.40, *p* < 0.01]. The second-order interaction was not observed [*F*_(8, 21, 573)_ = 0.93, *p* = 0.49]. The result revealed that mean F0, speech rate, and SD F0 influence the willingness to buy. Thus, *H*_1_ was supported. High mean F0, fast speech rate, or large SD F0 tended to increase the willingness to buy.

**Table 2:** Evaluation values used in analysis.

**Variable**	**Mean**	**SD**
Pleasure	4.01	1.29
Arousal	3.76	1.29
Dominance	3.94	1.06
Willingness to buy	3.88	1.24

### 4.2. Path analysis

**Figure 5:**
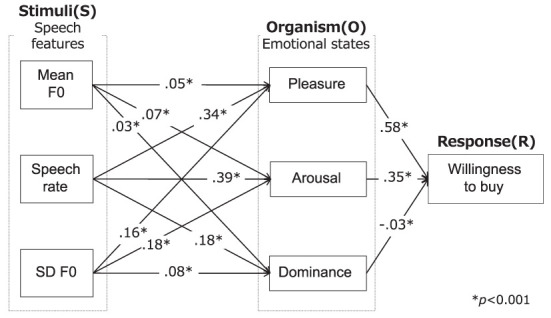
Results of path analysis.

Path analysis (Duncan, 1966) was conducted to examine direct dependencies between speech features and emotional states and between emotional states and the willingness to buy. Figure 5 shows a path diagram with a path coefficient. Some fit indices in Table 3 indicate that the model is valid. As shown in Figure 5, all speech features had a significant positive effect on all dimensions of the emotional states. Thus, *H*_2*a*_ was supported. The speech rate had a greater path coefficient than other speech features. For the effect of the emotional states on the willingness to buy, all emotional-state dimensions were significant. Therefore, *H*_2*b*_ was supported.

**Table 3:** Goodness-of-fit indices for path analysis.

**Fit indices**	**Accepted value**	**Value**
χ^2^ (Chi-square)		121.13
df		6
GFI	>0.9	0.999
AGFI	>0.9	0.997
CFI	< 0.10	0.997
RMSEA	< 0.10	0.003

GFI, AGFI, CFI, and RMSEA, respectively, indicate goodness of fit index, adjusted GFI, comparative fit index, and root mean square error of approximation.

### 4.3. Mediation analysis

**Figure 6:**
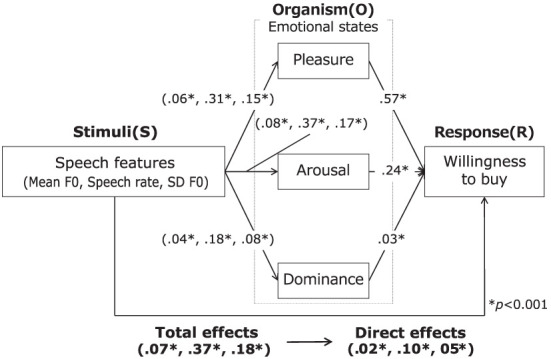
Results of mediation analysis.

Figure 6 and Table 4 show the results of mediation analysis. The mediating effects of PAD on the willingness to buy were all significant because the 95% confidence interval did not include zero. The largest mediating effect was pleasure, followed in order by arousal and dominance. The total and direct effects of speech features on the willingness to buy were both significant. These results indicate that PAD have a partial mediating effect on the relationship between speech features and the willingness to buy and that the emotion-mediated model is applicable to advertising speech. This supports hypothesis *H*_3_.

**Table 4:** Mediating effect of emotional states.

	**Pleasure**	**Arousal**	**Dominance**
Estimates	1.52	0.75	0.04
SE	0.05	0.03	0.01
CI.Lower	1.42	0.69	0.02
CI.Upper	1.63	0.82	0.06

## 5. Moderating effects of listener's gender and age group

The results of the moderated mediation analysis by listeners' gender are shown in Table 5. In an outcome of emotional states, all speech features had significant positive effects on all emotional states, and the product of speech rate and gender and that of SD F0 and gender for pleasure and arousal had significant positive effects. In an outcome of the willingness to buy, all speech features and emotional states had significant positive effects on the willingness to buy. The product of pleasure and gender and that of dominance and gender had significant positive effects, and that of arousal and gender had significant negative effects.

**Table 5:** Results of moderated mediation analysis for listeners' gender.

**Outcome**	**Speech features**→**Emotional states**									**Emotional states**		
	**Pleasure**			**Arousal**			**Dominance**			→**Willingness to buy**		
	*B*	*SE*	*t*	*B*	*SE*	*t*	*B*	*SE*	*t*	*B*	*SE*	*t*
Mean F0	0.32^**^	0.03	9.49	0.40^**^	0.03	12.03	0.17^**^	0.03	5.65	0.08^**^	0.02	3.69
Speech rate	0.62^**^	0.01	49.0	0.73^**^	0.01	59.77	0.30^**^	0.01	27.37	0.19^**^	0.01	21.65
SD F0	1.86^**^	0.08	23.79	2.09^**^	0.08	27.48	0.82^**^	0.07	12.13	0.59^**^	0.05	12.31
Gender	0.18^**^	0.02	10.67	0.07^**^	0.02	4.06	–0.04^**^	0.01	–2.75	–0.37^**^	0.04	–8.26
Mean F0 × Gender	0.05	0.07	0.70	0.12	0.07	1.71	0.04	0.06	0.71			
Speech rate × Gender	0.07^**^	0.03	2.84	0.16^**^	0.03	6.49	0.04	0.02	1.58			
SD F0 × Gender	0.32^*^	0.16	1.98	0.70^**^	0.16	4.45	0.21	0.14	1.54			
Pleasure										0.55^**^	0.01	72.73
Arousal										0.24^**^	0.01	30.15
Dominance										0.03^**^	0.01	4.49
Pleasure × Gender										0.05^**^	0.02	3.33
Arousal × Gender										–0.04^**^	0.02	–2.36
Dominance × Gender										0.06^**^	0.01	4.26
*F*	448.9			642.5			133.1			4409		
*R* ^2^	0.13^**^			0.17^**^			0.04^**^			0.67^**^		

Columns 2–4 and 5 show moderating effects of gender on emotional states and on willingness to buy, respectively.

*p < 0.05 and

**p < 0.01.

Table 6 shows the results of the conditional mediating effect of emotional states when gender moderated between speech features and emotion, and between emotional states and the willingness to buy. All mediating effects of emotional states were significant for female listeners. For male listeners, the mediating effects of pleasure and arousal were significant, but the mediating effect of dominance was not significant because the 95% confidence interval included zero. The mediating effect of all emotional states tended to be larger for female listeners than for male listeners. Thus, *H*_4_ was supported.

**Table 6:** Conditional mediating effect of emotional states when gender moderated between speech features and emotional states, and between emotional states and willingness to buy.

	**Pleasure**				**Arousal**				**Dominance**			
	**Estimates**	**SE**	**95%CI**		**Estimates**	**SE**	**95%CI**		**Estimates**	**SE**	**95%CI**	
			**Lower**	**Upper**			**Lower**	**Upper**			**Lower**	**Upper**
Male	1.40	0.06	1.28	1.52	0.72	0.04	0.64	0.79	0.01	0.01	–0.01	0.03
Female	1.77	0.08	1.61	1.94	0.80	0.06	0.69	0.92	0.09	0.02	0.06	0.12
Not-moderated	1.52	0.05	1.42	1.63	0.75	0.03	0.69	0.82	0.04	0.01	0.02	0.06

The results of the moderated mediation analysis by listeners' age group are shown in Table 7. In an outcome of emotions, all speech features had significant positive effects on all emotional states. The product of speech rate and age group for all emotional states and that of SD F0 and age group in arousal and dominance had significant positive effects. In an outcome of willingness to buy, all speech features and emotional states had significant positive effects on the willingness to buy. The product of pleasure and age group had significant positive effects, and that of arousal and age group and that of dominance and age group had significant negative effects on the willingness to buy.

**Table 7:** Results of moderated mediation analysis for listeners' age.

**Outcome**	**Speech features**→**Emotional states**									**Emotional states**		
	**Pleasure**			**Arousal**			**Dominance**			→**Willingness to buy**		
	*B*	*SE*	*t*	*B*	*SE*	*t*	*B*	*SE*	*t*	*B*	*SE*	*t*
Mean F0	0.32^**^	0.03	9.48	0.40^**^	0.03	12.01	0.17^**^	0.03	5.66	0.07^**^	0.02	3.65
Speech rate	0.62^**^	0.01	48.88	0.73^**^	0.01	59.69	0.30^**^	0.01	27.41	0.18^**^	0.01	22.79
SD F0	1.85^**^	0.08	23.54	2.08^**^	0.08	27.34	0.82^**^	0.07	12.20	0.60^**^	0.05	12.80
Age group	–0.01	0.01	–1.24	0.02^**^	0.01	2.79	0.00	0.01	–0.36	0.00	0.00	–1.30
Mean F0 × Age group	0.00	0.02	–0.09	–0.03	0.02	–1.31	–0.04	0.02	–1.77			
Speech rate × Age group	0.04^**^	0.01	4.70	0.03^**^	0.01	3.25	0.05^**^	0.01	6.14			
SD F0 × Age group	0.05	0.06	0.87	0.11^*^	0.05	2.02	0.26^**^	0.05	5.39			
Pleasure										0.56^**^	0.01	104.38
Arousal										0.22^**^	0.01	38.56
Dominance										0.03^**^	0.01	5.34
Pleasure × Age group										0.06^**^	0.01	15.15
Arousal × Age group										–0.03^**^	0.01	–6.22
Dominance × Age group										–0.01^**^	0.01	–3.18
*F*	432.4			632.9			141.7			4454		
*R* ^2^	0.12^**^			0.17^**^			0.04^**^			0.67^**^		

Columns 2–4 and 5 show moderating effects of age on emotional states and on willingness to buy, respectively.

*p < 0.05 and

**p < 0.01.

Table 8 shows the results of the conditional mediating effect of emotional states when the age group moderated between speech features and emotional states, and between emotional states and the willingness to buy. This comparison was conducted at three levels of the listener's age group: mean -1SD (younger), mean, and mean +1SD (older). The mediating effect of pleasure was significant in all conditions and tended to be larger as listeners got older. The mediating effect of arousal was significant under all conditions and tended to be smaller as listener age increased, unlike pleasure. The mediating effect of dominance was significant for young and mean age but did not differ. For older, the mediating effect of dominance was not significant because the 95% confidence interval included zero. This suggests that speech that enhances pleasure in older people and arousal in younger people may increase their willingness to buy. Thus, *H*_5_ was supported.

**Table 8:** Conditional mediating effect of emotional states when age moderated between speech features and emotional states and between emotional states and willingness to buy.

	**Pleasure**				**Arousal**				**Dominance**			
	**Estimates**	**SE**	**95%CI**		**Estimates**	**SE**	**95%CI**		**Estimates**	**SE**	**95%CI**	
			**Lower**	**Upper**			**Lower**	**Upper**			**Lower**	**Upper**
Mean – 1SD	1.31	0.05	1.20	1.41	0.78	0.05	0.69	0.88	0.04	0.01	0.02	0.06
Mean	1.56	0.05	1.45	1.66	0.72	0.03	0.66	0.78	0.04	0.01	0.02	0.06
Mean + 1SD	1.82	0.06	1.70	1.95	0.64	0.04	0.57	0.73	0.02	0.02	–0.01	0.06
Not-moderated	1.52	0.05	1.42	1.63	0.75	0.03	0.69	0.82	0.04	0.01	0.02	0.06

Mean – 1SD, Mean, and Mean + 1SD are 25, 39, and 53 years old, respectively.

## 6. Discussion

This study was mainly motivated to investigate whether SOR theory mediated by emotional states can explain the willingness to buy from advertising speech. From the results of ANOVA, we found that speech features of mean F0, speech rate, and SD F0 affected the willingness to buy (*H*_1_). Path analysis confirmed that there was a dependency between speech features and emotional states and between emotional states and the willingness to buy (*H*_2*a*_ and *H*_2*b*_). Through mediation analysis, we found that there was a mediating effect of emotional states between speech features and the willingness to buy (*H*_3_). These results suggest that SOR theory using the PAD model can explain the willingness to buy from advertising speech.

The result that fast-speech rate increases the willingness to buy is consistent with Chattopadhyay (Chattopadhyay et al., 2003)'s results. However, our results were not consistent with their finding of increased willingness to buy at a low mean F0 (Chattopadhyay et al., 2003) or with the finding of no correlation between mean F0 and SD F0 of salespeople and sales performance by Peterson et al. (1995). One reason for this difference could be that their emotional states were different. Another possible reason is the difference in experimental methods between speech conversion in this study and speech analysis of multiple salespeople in Peterson et al.'s study.

Rosenberg et al. examined the relationship between the rating of some speakers' charisma for political statements and several speech features (Rosenberg and Hirschberg, 2009). They reported that a higher mean F0, greater SD F0, and faster speech rate were rated more charismatic, which is the same trend as in our study. Although the rating of charisma is a different task from the willingness to buy, the reason the results indicate the same trend may be due to the unconscious response of wanting to buy products from trustworthy persons.

As a result of the path analysis, the path coefficients from speech rate to emotional states and from pleasure to the willingness to buy were the largest. These results are consistent with previous studies (Tursunov et al., 2019; Anwar et al., 2020; Nagano et al., 2021).

The results of the mediation analysis showed that emotional states had a partial mediating effect because the significance of the direct effect of the speech features was observed. If the mediating effect of the emotional states was sufficient as a mediator, the significance of the direct effect should be lost. Thus, there are other mediators besides emotional states regarding the effect of speech features on the willingness to buy. Another possible mediating factors is impressions of speech. It may be effective to subdivide the relationship between speech features and emotional states (Li and Akagi, 2018).

The emotional mediating effects on the willingness to buy from advertising speech may differ depending on the attributes of the listeners. We analyzed the moderating effects of the listener's gender and age group on emotional states and the willingness to buy.

In moderated mediation analysis, the mediating effects of all emotional states differed depending on the listeners' gender (*H*_4_). It tended to be larger for female listeners than for male listeners. This may be related to the fact that females have better emotional perception than males (Bonebright et al., 1996; Kring and Gordon, 1998; Keshtiari and Kuhlmann, 2016).

From the results for the listeners' age group, the mediating effects of all emotional states, in particular, pleasure and arousal, differed depending on the listeners' age group (*H*_5_). The mediating effect of pleasure tended to be larger as listeners age increased, but the mediating effect of arousal tended to be smaller. Schmidt et al. compared the emotional recognition ability for various speakers' emotional voices between younger and older adults (Füllgrabe et al., 2015). They reported that older adults were more likely than younger adults to respond to differences in mean F0 cueing pleasure and less strongly to intensity differences cueing arousal. The results of our study are similar. It is also possible that hearing loss in older adults may have affected the results, but further investigation is required.

When the moderating effects of listeners' gender and age group were taken into account, the mediating effect of dominance for male and older were not significant. The significance of dominance is still controversial, with many studies indicating there is no significant difference (Donovan and Rossiter, 1982; Donovan et al., 1994), and others indicating there is Yalcha and Spangenberg (2000). The effect of dominance may be more likely affected by individual characteristics such as listeners' attributes.

The finding that emotional states mediate the willingness to buy from advertising speech and that the attributes of the listener moderate the mediating effect suggests that salespeople may improve sales by speaking in a way that appeals to the customer's emotional states. However, our study had several other limitations. Since the experiment was conducted *via* crowdsourcing, the listening environment (e.g., device, intensity, background noise) of the participants was not controlled. These differences in the experiment may have influenced the results.

Another problem is that the types of speech features, sentences, the domain of advertisement, and speakers were limited, and participants were not required to buy the products promoted within the advertising speech. To confirm whether the results of this study are generally applicable, experiments with more speakers, items, or sentences and participants' real purchasing behavior should be conducted (Joo et al., 2020).

## 7. Conclusion

We analyzed the relationship between speech features, emotional states (pleasure, arousal, dominance), and the willingness to buy on the basis of a consumer-behavior model. Large-scale subjective evaluation data were collected *via* crowdsourcing. The participants listened to speech with different speech features (mean F0, speech rate, or SD F0) and rated their willingness to buy the products advertised in the speech and their perceived emotional states. The results of a three-way ANOVA indicate that speech features affect the willingness to buy. As the result of path and mediation analyzes, the emotional states were revealed to function as a partial mediator regarding the influence of speech features on the willingness to buy, and the emotion-mediated model is effective. In particular, increasing pleasure and arousal can be expected to enhance the willingness to buy. The moderating effects of the listener's gender and age group on emotional states and the willingness to buy were also analyzed. The mediating effects of all emotional states tended to be larger for female listeners than for male listeners. From the results of the listeners' age group, the mediating effect of pleasure tended to be larger as listener age increased, but the mediating effect of arousal tended to be smaller. For future work, we will investigate whether the same tendency is observed when speech sentences or speakers are changed.

## Data availability statement

The original contributions presented in the study are included in the article/Supplementary material, further inquiries can be directed to the corresponding author.

## Ethics statement

Ethical review and approval were not required for this study in accordance with the national legislation and institutional requirements. Written informed consent for participation was not required in accordance with national legislation and institutional requirements.

## Author contributions

MN and YI conceived the study and carried out the experiment. MN, YI, and SH carried out the modeling and analytical investigations. All authors wrote the paper and reviewed the manuscript and contributed to the article and approved the submitted version.
