# Supplementary material for: Perceived emotional states mediate willingness to buy from advertising speech
Source: Front Psychol. 2023 Jan 9;13:1014921. doi: 10.3389/fpsyg.2022.1014921 (PMC9870619; doi:10.3389/fpsyg.2022.1014921)
Supplement: Supplementary file 1 [file Data_Sheet_1.docx]

The sentences used in the experiment are as follows.

- Mitsubishi Electric’s “Kirigamine Move-Eye” is able to monitor temperature fluctuations in extent living spaces and control airflow.
- This washing machine has an “Ag+ Ion-Coating” that prevents static electricity and unpleasant odors. This machine uses less water to wash clothes with its “powerful slamming wash” and “circulating washing system”.
- This refrigerator is ranked No. 1 in the refrigerator category. It can be freely placed in any location, thanks to its “both side opening hinge” design.
- This PC is equipped with a high-definition 20-inch wide screen, so you can enjoy amazingly beautiful pictures. It is also equipped with the latest OS and is an integrated, easy-to-use PC.
